# Fructosamine is a valuable marker for glycemic control and predicting adverse outcomes following total hip arthroplasty: a prospective multi-institutional investigation

**DOI:** 10.1038/s41598-021-81803-6

**Published:** 2021-01-26

**Authors:** Noam Shohat, Karan Goswami, Leigham Breckenridge, Michael B. Held, Arthur L. Malkani, Roshan P. Shah, Ran Schwarzkopf, Javad Parvizi

**Affiliations:** 1 Rothman Orthopaedic Institute at Thomas Jefferson University, 125 S 9th St. Ste 1000, Philadelphia, PA 19107 USA; 2 Sackler Faculty of Medicine, Tel Aviv University, Ramat Aviv, Israel; 3 Department of Orthopaedic Surgery, Columbia University, New York, NY USA; 4 Department of Orthopaedics, University of Louisville, Louisville, KY USA; 5 Department of Orthopaedic Surgery, New York University, New York, NY USA

**Keywords:** Medical research, Orthopaedics

## Abstract

Recently, fructosamine has shown promising results in predicting adverse outcomes following total knee arthroplasty. The purpose of this study was to assess the utility of fructosamine to predict adverse outcomes following total hip arthroplasty (THA). A prospective multi-center study involving four institutions was conducted. All primary THA were evaluated for glycemic control using fructosamine levels prior to surgery. Adverse outcomes were assessed at a minimum 1 year from surgery. Primary outcome of interest was periprosthetic joint infection (PJI) based on the International Consensus Meeting (ICM) criteria. Secondary outcomes assessed were superficial infections, readmissions and death. Based on previous studies on the subject, fructosamine levels above 293 µmol/L were used to define inadequate glycemic control. Overall 1212 patients were enrolled in the present study and were available for follow up at a minimum 1 year from surgery. Of those, 54 patients (4.5%) had elevated fructosamine levels (> 293 µmol/L) and these patients were 6.7 times more likely to develop PJI compared to patients with fructosamine levels below 293 µmol/L (p = 0.002). Patients with elevated fructosamine were also associated with more readmissions (16.7% vs. 4.4%, p < 0.007) and a higher mortality rate (3.7% vs. 0.6%, p = 0.057). These associations remained statistically significant in a multi-regression analysis after adjusting for age, comorbidities and length of stay; Adjusted odds ratio were 6.37 (95% confidence interval 1.98–20.49, p = 0.002) for PJI and 2.68 (95% confidence interval 1.14–6.29, p = 0.023) for readmissions. Fructosamine is a good predictor of adverse outcomes in patients undergoing THA and should be used routinely to mitigate morbidity and mortality risk.

## Introduction

Much attention has been devoted in recent years to develop strategies that can mitigate the risk of adverse outcomes following total joint arthroplasty (TJA), with a special emphasis to strategies that can reduce periprosthetic joint infection (PJI)^1,2^. Among the risk factors known to affect outcome of any surgical procedure, including TJA, is proper glycemic control^1,3–5^. The latter is ever more important as up to 50% of patients undergoing THA were found to have hyperglycemia^6,7^. Moreover, glycemic control is considered one of the only modifiable factors, the control of which is demonstrated to reduce the risk of complications^8,9^.

While it is well acknowledged that hyperglycemia is associated with adverse outcomes following surgery, there remains controversy about which glycemic markers to use and what interval from surgery they are most predictive^1,4^. In the preoperative period, 3 month glycemic control as measured by glycated hemoglobin (HbA1c) has been used, whereas in the perioperative period fasting glucose is routinely used for risk stratification^10–13^ Many institutions now use predetermined cutoffs values for both fasting glucose and HbA1c, and postpone surgery to patients with elevated levels of one or both of these glycemia markers. The effectiveness of this protocol in reducing complications is yet to be proven^14^.

Fructosamine is not a new glycemic marker^15^. The monitoring of glycated protein (mainly albumin) was introduced 40 years ago and has recently been revisited as a tool for preoperative screening due to clear advantages in the preoperative period^16,17^. The fact that fructosamine represents sugar levels over a 2–3 week period makes it a valuable marker for both screening and monitoring therapeutic interventions. If patients with inadequate glycemia control were to have elective surgery postponed based on elevated fructosamine levels, return to better glycemic control could be confirmed within 2 weeks using fructosamine as opposed to 3 months using HbA1c.

In a recent study, fructosamine was shown to be more valuable than HbA1c in predicting early complications following TKA^16^. The purpose of this prospective multicenter study was to evaluate the role of fructosamine in predicting 1-year complications in patients undergoing elective THA.

## Materials and methods

Following IRB approval by the ethics committee of Thomas Jefferson University Hospital (IRB-16D.225), NYU Langone (IRB-i18-00017), Columbia University (IRB-AAAR7050), and University of Louisville (IRB-17.0813), these four academic centers participated in this prospective study. Written informed consent was obtained from all participants of this study. All methods were carried out in accordance with the relevant guidelines and regulations based on the approvals. During the time period between August 2017 and November 2018, all elective primary THA patients performed by 8 fellowship trained surgeons were investigated for glycemic control using fructosamine, HbA1c and fasting plasma glucose (FPG) levels within four weeks prior to surgery. Postoperative day 1 (POD) morning glucose levels were also captured. Patients were followed-up for a minimum of 1 year.

### Data collection

Demographic and perioperative factors that could serve as confounders were collected and are presented in Table 1. Obesity was defined as body mass index (BMI) above 30 kg/m^2^. Charlson Comorbidity Index (CCI) was calculated for each patient based on medical records. Special attention was given to diabetic status and was collected both from self-reported questionnaires, as well as from medical records. Diabetes was considered complicated if systemic complications were present.

**Table 1:** Demographic characteristics and comorbidities of the entire cohort and differences between those with fructosamine levels below and above 293 µmol/L.

	Overall	Fructosamine < 293 µmol/L (n = 1158)	Fructosamine ≥ 293 µmol/L (n = 54)	p value
Age, year	63.97 (11.39)	63.68 (11.33)	70.12 (11.16)	< 0.001
Sex (female)	616 (50.8%)	594 (51.3%)	22 (40.7%)	0.163
Operated hip (right)	573 (48.1%)	547 (47.2%)	26 (48.1%)	0.797
Body-mass index (kg/m^2^)	29.07 (5.40)	29.10 (5.43)	28.32 (4.64)	0.305
Obese (> 30 kg/m^2^)	457 (38.4%)	437 (38.4%)	20 (37.7%)	1.000
Charlson Comorbidity Index	0.52 (1.00)	0.49 (0.94)	1.24 (1.60)	0.001
Score ≥ 3	63 (5.2%)	55 (4.75%)	8 (14.81%)	< 0.001
History of diabetes mellitus	111 (9.2%)	90 (7.8%)	21 (38.9%)	< 0.001
With complications	9 (7.4%)	5 (0.4%)	4 (7.4%)	< 0.001
Length of stay (days)	1.51 (1.40)	1.49 (1.40)	2.00 (1.44)	0.009

Data are presented as mean values (SD) or as numbers (%).

*Excluding diabetic element of scoring.

The primary outcome assessed was PJI based on the 2018 International Consensus Meeting (ICM) definition^18^. Secondary adverse outcomes included superficial wound complications, hospital readmissions for causes related to their surgery (including deep vein thrombosis and prosthesis-related complications) and death. A wound complication was predefined and included more than 1 week of drainage, postponed hospital discharge due to wound issues, the use of antibiotics, or return to the operating room for wound care.

### Statistical analysis

A power analysis was performed based on prior literature and the rate of PJI stratified based on fructosamine levels (above and below 293 µmol/L) was predicted to be approximately 1:3.5. Sample size was projected supposing a 2-sided t-test with an alpha level of 0.05 and 0.8 power. A sample size of 1100 patients was deemed appropriate. Inadequate glycemic control was assumed in patients with fructosamine levels above 293 µmol/L (4) For verification, receiver operating characteristic (ROC) analysis was performed and the area under the curve (AUC) was calculated for fructosamine and the risk for PJI. Adverse outcomes were assessed following glycemic control stratification. Categorical variables were analyzed using the Chi square test and continuous variables by the Student’s t-test. A p value < 0.05 was considered significant except for death, where a p value < 0.1 was set to be considered statistically significant due to its gravid impact. All demographic, comorbidity and hospitalization variables that were significantly different between those with high (above 293 µmol/L) and normal (below 293 µmol/L) fructosamine were included in a logistic regression analysis, and adjusted odds ratios were calculated. Death was not assessed in a regression analysis due to the minor incidence. A scatter plot was created to assess the association between fructosamine and HbA1c levels. A subgroup analysis of patients with FPG levels above 100 mg/dL was also performed to assess the utility of fructosamine in a more specific cohort.

## Results

**Figure 1:**
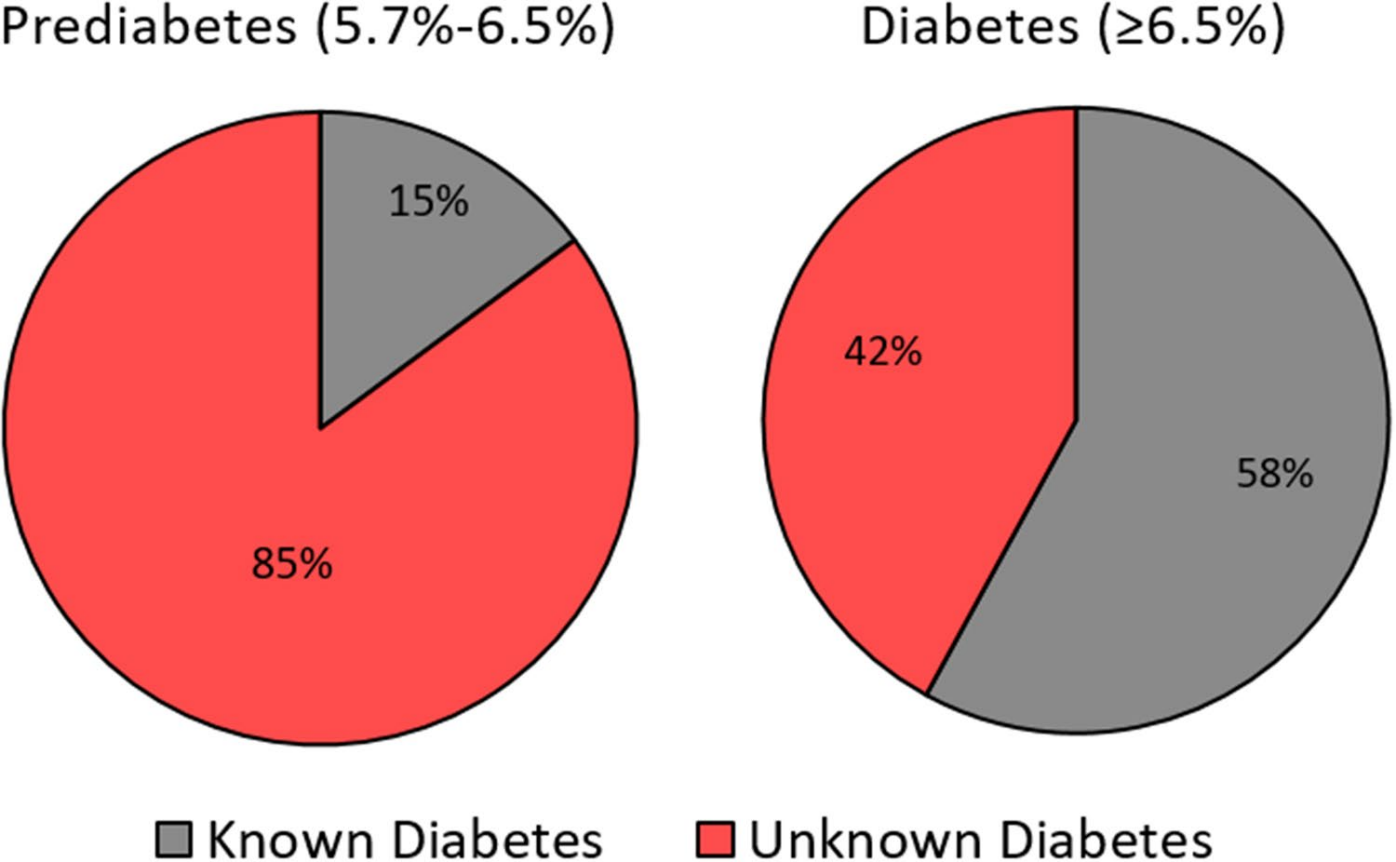
Proportion of patient with known diabetes with HgbA1c levels in the prediabetes and diabetes range.

During the study period a total of 1,212 patients (616 females) underwent THA who had preoperative fructosamine collected and were available for 1-year follow-up after their primary THA. Mean age was 63.9 years (standard deviation [SD] 11.39). Overall, 111 patients (9.2%) were known to have diabetes. Screening all THA patients using HbA1c revealed that many patients were unaware of their diabetic state. Of the 303 patients who had HbA1c levels in the prediabetes range (5.7–6.5%) upon screening, only 45 (15%) were previously diagnosed with diabetes (i.e. 85% were unaware of their pre-diabetic state), and of the 88 patients with HbA1c levels in the diabetic range (≥ 6.5%), only 51 patients (58%) had a prior diagnosis of diabetes (i.e. 42% were unaware they had diabetes) (Fig. 1).

Mean fructosamine levels were 241.91 µmol/L (range, 108 to 490) for the entire cohort. In 54 patients (4.5%) fructosamine levels were greater than 293 µmol/L indicating inadequate glycemic control, however only 21 of them (38.9%) had a prior diagnosis of diabetes. Compared to patients with fructosamine levels below 293 µmol/L, patients with levels greater than 293 µmol/L were older (p < 0.001), had more comorbidities (p = 0.001) including diabetes (p < 0.001) and had a longer hospital stay (p = 0.009), as presented in Table 1.

**Figure 2:**
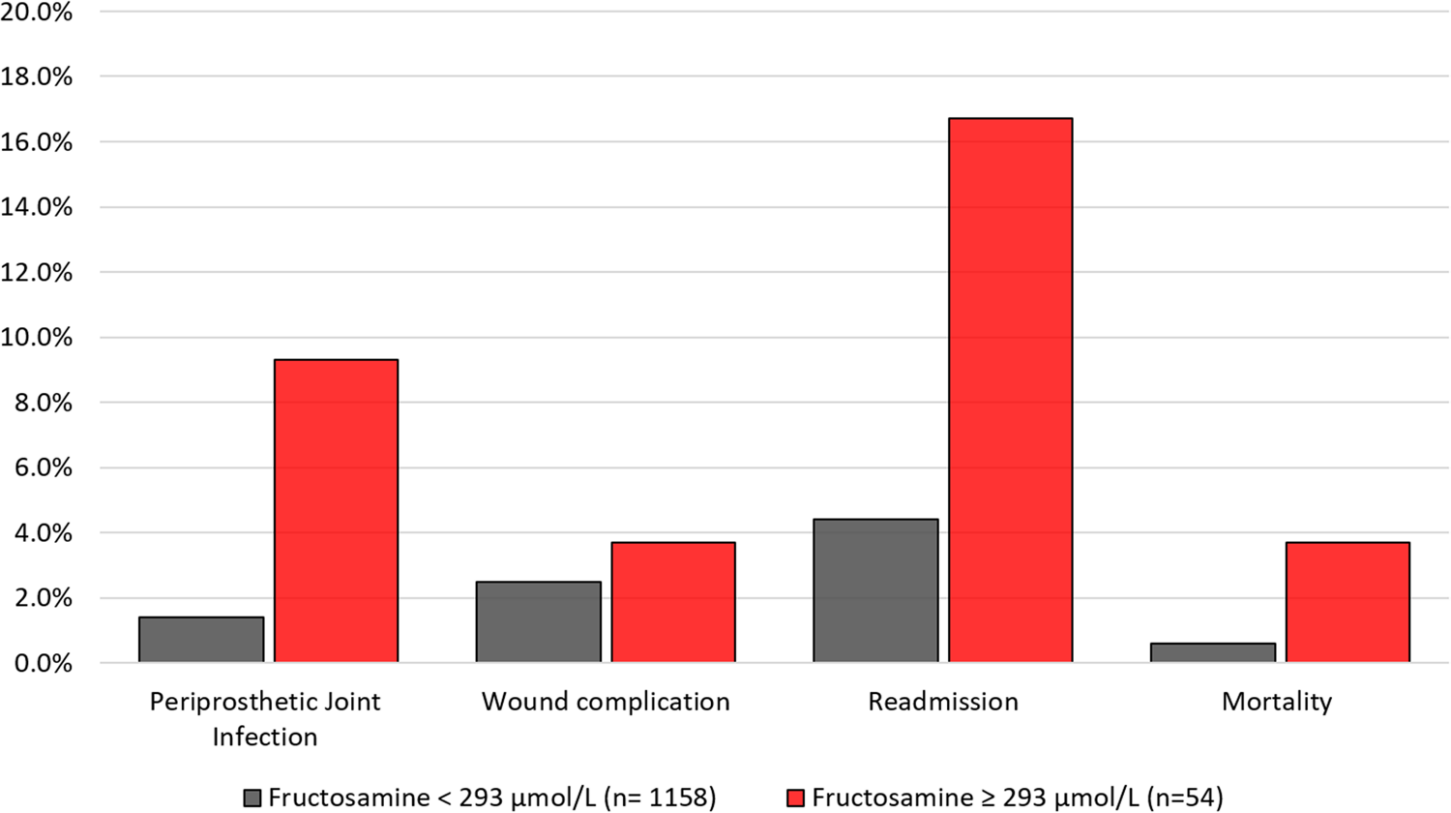
Complication rates stratified based on fructosamine levels below 293 μmol/L (n = 1158) and above 293 μmol/L (n = 54). *Represents statistically significant differences.

High fructosamine levels were associated with PJI (AUC 0.58, 95% Confidence Interval 0.40–0.70]. Fructosamine levels above 293 µmol/L were significantly associated with PJI, readmissions and death (Table 2, Fig. 2). PJI rates were 6.64 times higher (p = 0.002), readmission rates were 3.80 times higher (p = 0.001), and mortality was 6.17 times higher (p = 0.057) in patients with elevated fructosamine levels. Superficial wound complication rates were similar between those with fructosamine levels above and below 293 µmol/L (3.7% versus 2.5%, p = 0.645). After adjusting for age, comorbidities and length of stay which were all significantly (p < 0.05) higher in those with fructosamine above 293 µmol/L, the above associations with PJI (adjusted odds ratio 6.37, 95% confidence interval 1.98–20.49; p = 0.002) and readmissions (adjusted odds ratio 2.68, 95% confidence interval 1.14–6.29; p = 0.023) remained statistically significant.

**Table 2:** Adverse outcomes in patients with high (≥ 293 µmol/L) and low (< 293 µmol/L) fructosamine levels in the entire cohort and in a subgroup who had glucose levels above 100 mg/dL.

Complications	Total cohort (n = 1212)			Fasting plasma glucose ≥ 100 mg/dL (n = 423)		
	Normal fructosamine (n = 1158)	High fructosamine (n = 54)	p value	Normal fructosamine (n = 391)	High fructosamine (n = 32)	p value
PJI	16 (1.4%)	5 (9.3%)	0.002	6 (1.5%)	4 (12.5%)	0.004
Wound complication	29 (2.5%)	2 (3.7%)	0.645	14 (3.6%)	2 (6.3%)	0.345
Readmission	51 (4.4%)	9 (16.7%)	0.001	16 (4.1%)	7 (21.9%)	0.001
Mortality	7 (0.6%)	2 (3.7%)	0.057	4 (1.0%)	1 (3.1%)	0.327

*PJI* periprosthetic joint infection.

In the subgroup of patients with FPG above 100 mg/dL (n = 423), a higher proportion of patients had fructosamine levels above 293 µmol/L (7.6%). Four of the 5 PJI cases, 7 of the 9 readmissions and 1 of the 2 deaths that were captured in those with elevated fructosamine had occurred in patients with FPG above 100 mg/dL. As before, associations with PJI and readmissions were significantly higher in those with fructosamine levels above 293 µmol/L (Table 2).

**Figure 3:**
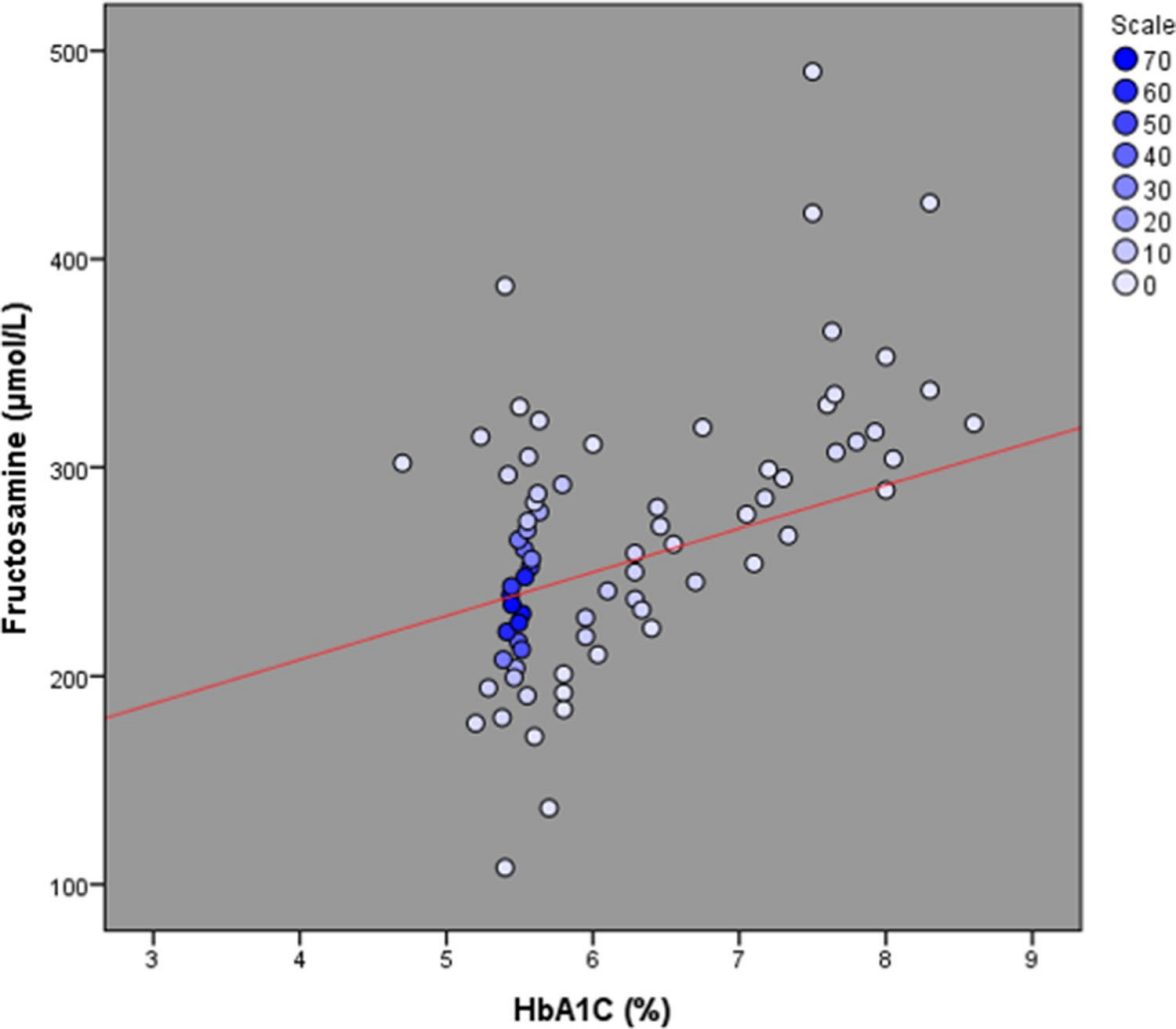
A scatter plot correlating HbA1c and fructosamine (R^2^ = 0.18).

There was a positive correlation between fructosamine and HbA1c levels (Fig. 3). Of the patients who had fructosamine levels greater than 293 µmol/L, 39 patients (72.2%) were in the prediabetes or diabetes range based on HbA1c levels. Fructosamine levels greater than 293 µmol/L were significantly associated with higher FPG, and higher POD-1 glucose levels (Table 3). However, among the 5 patients with fructosamine greater than 293 µmol/L who developed PJI, only 2 had a diagnosis of diabetes. HbA1c levels were less than 7.5% in 2 of these patients (40%) and less than 8% in 4 of them (80%). On the contrary, there were no patients with HbA1c levels above 7.5% or 8.0% that did not have fructosamine levels above 293 µmol/L.

**Table 3:** Data on glycemic control in patients with fructosamine levels below and above 293 µmol/L.

Glycemic control	Overall	Fructosamine < 293 µmol/L (n = 1158)	Fructosamine ≥ 293 µmol/L (n = 54)	p value
Hemoglobin A1c, %	6.15 (15.67)	6.12 (16.1)	6.71 (1.33)	0.788
5.7% to 6.5% (prediabetes)	303 (30.0%)	293 (30.6%)	10 (18.9%)	< 0.001
≥ 6.5% (diabetes)	88 (8.7%)	59 (6.2%)	29 (54.7%)	< 0.001
≥ 7.5%	30 (3.0%)	10 (1.0%)	20 (37.7%)	< 0.001
Preoperative fasting glucose (mg/dL)	100.61 (24.39)	99.11(20.82)	133.88 (55.53)	< 0.001
≥ 100 (mg/dL)	423 (37.9%)	391 (36.6%)	32 (66.7%)	< 0.001
Postoperative day 1 glucose	137.51 (32.42)	135.32 (29.14)	171.67 (55.09)	< 0.001

Data are presented as mean values (SD) or as numbers (%).

*Excluding diabetic element of scoring.

## Discussion

The main finding of this prospective study was that fructosamine, as a glycemic marker, is an excellent predictor of adverse outcomes following THA. Another important, and sobering, finding of the study, that corroborates prior studies^6,7^, is that a large proportion of patients undergoing elective THA appear to be hyperglycemic or undiagnosed diabetics who are unaware of their poor glycemic control. In a recent International Consensus Meeting (ICM), the issue of screening all patients for hyperglycemia prior to surgery was discussed with 76% of delegates voted in favor of such protocol^19^. The rationale behind such recommendation is that by evaluating glycemic control only in patients with known diabetes, we are neglecting a large cohort of patients with unrecognized hyperglycemia and/or diabetes that are at increased risk for complications^20^. The major question that remains is which agent best represents the glycemic control of a patient.

HbA1c remains the gold standard for assessing glycemic control and is invaluable in the treatment of diabetic patients. It is a predictor of diabetic complications, and interventions that lower HbA1c levels have proved themselves useful in reducing the risk of complications^21^. Whereas the overall utility of HbA1c is beyond question, discordances between HbA1c and other clinically reliable biochemical measures of glycemic control such as fructosamine are commonly encountered^22^. These discordances are mainly attributed to the time frame over which HbA1c equilibrates compared with shorter-term measures of plasma glucose^23–25^. It has also been hypothesized that discordances between HbA1c and other measures of glycemia such as fructosamnie are due to physiologic processes consistent within individuals over time and these variations by them self may have a role in predicting complications^26^. Our finding of elevated fructosamone levels in patients without a diagnosis of diabetes or elevated HbA1c levels is in accordance with the above mentioned discordances and reinforces recent findings in arthroplasty patients^16,17,27^.

It has become common practice to evaluate the preoperative HbA1c level in patients with diabetes prior to surgery and postpone surgery in patients with elevated levels^19^. The American Diabetes Association (ADA) states that HbA1c > 7% is representative of poor glycemic control^28^. In a recent multicenter study on patients undergoing TJA, HbA1c > 7.7% was found to be associated with higher rate of PJI and other complications^10^. Based on the latter study, our institution and many others will cancel elective orthopedic surgery in patients with HbA1c > 7.5 or 8%^4,11,29^. While the rationale behind such practice appears to be logical, the value of this protocol in reducing postoperative complications has not been proven. Further, HbA1c represents glycemic control over the 120 day life cycle of red blood cells. Thus, patients with elevated HbA1c who had their elective surgery cancelled may need to wait for 3 months before confirming that implementation of better glycemic control was effective and be able to undergo surgery. Another glycemic marker that has proven to be valuable is fasting blood glucose^4^. The Center for Disease Control (CDC) in their recent guidelines for prevention of surgical site infection states that patients with fasting glucose of 200 mg/dL have a poor glycemic control and should not undergo elective surgical procedures^1^. Though valuable, there are logistic issues associated with measuring fasting glucose levels in patients awaiting elective surgery. Further, fasting glucose levels are under immense influence of numerous factors including stress, which most patients undergoing surgery would exhibit^30,31^.

Fructosamine is a measure of glycated protein in the plasma, with albumin being the major constituent, and thus reflects glycemic control over the proceeding 2–3 weeks^32^. In daily diabetes care unrelated to surgery, fructosamine adds little to the commonly used HbA1c, as majority of literature pertaining to screening and treatment is on the latter. In the preoperative period, fructosamine has a substantial advantage as a glycemic marker as it allows one to monitor response to treatment in a much shorter time frame compared to HbA1c. The current study adds to the body of literature that shows fructosamine is a valuable screening tool for hyperglycemia in patients undergoing elective surgery^16,17^. There is a direct correlation between fructosamine and HbA1c in patients with and without diabetes, as seen in this study. While a correlation between the two glycemic markers exists, there are important differences, mostly related to the time frame of glycemic control that each of these markers represent. There are variations in institutional policies regarding the optimal threshold for HbA1c, with most institutions using HbA1c between 7.5 and 8.0% as representative of poor glycemic control^4^. The current study demonstrates that if such thresholds for HbA1c were to be used, instead of the fructosamine levels, many patients who developed PJI or had adverse outcome would have been missed.

This study adds to recent reports on the utility of fructosamine as a valuable glycemic marker, particularly in patients undergoing elective TJA^16,17^. The cost of fructosamine testing is negligible compared to the costs associated with complications following THA, and if it is indeed used in the preoperative period it may also be cos-beneficial. The cost of a single fructosamine test is $19.77 amounting to a total cost of $23,961 in the current study whereas the cost of treating a single PJI event is estimated as $100,000 per case^33^. The fact that 5 patients with high fructosamine levels developed PJI that could have been theoretically prevented, support the notion that all patients undergoing THA should be screened for glycemic control using fructosamine. However, we are aware that screening all patients prior to surgery may not be possible for many institutions. In an attempt to isolate a smaller group that would benefit from screening, we performed a sub-analysis of the current cohort and discovered that patients with fasting glucose > 100 mg/dL would most benefit from glycemic screening using fructosamine. Screening only those with high fasting glucose would reduce by 65% the number of patients that are screened, and still capture 80% of the patients that are at higher risk of postoperative complications. Interestingly, not only high fructosamine levels are informative, but normal levels are also beneficial in risk stratification. A normal fructosamine level in patients with FPG > 100 mg/dL can be reassuring for the treating physician as complication rates in this group were similar to those seen with FPG < 100 mg/dL. This strategy of screening may be an efficient compromise for certain centers.

While we cannot say for sure why exactly fructosamine is such a powerful prognostic marker, there are several theories. First, fructosamine reflects fluctuations in glucose which have been associated with complications following TJA^27,34,35^. Both in-vivo and in-vitro studies attribute the negative effects of these fluctuations to the activation of pro-inflammatory proteins and excessive oxidative stress^36^. Furthermore, short-term fluctuations in glucose levels may have a larger effect on inflammatory cytokine levels than continuous hyperglycemia and has been shown to affect all major components of innate immunity, namely decreased neutrophil chemotaxis and phagocytosis thus impairing the ability of the host to combat infection^37,38^. Second, it is possible that it is not the essence of diabetes itself, but the high sugar level close to surgery that increase susceptibility to surgical site infections. By reflecting glucose levels over the 2–3 weeks prior, fructosamine better reflects the glycemic control closer to surgery. Finally, fructosamine may be a more sensitive marker for glycemic disturbances in patients without diabetes^39^.

This study had several limitations. First and most importantly, this was an observational study and while the results suggest an association between fructosamine and complications, we cannot say for sure that controlling fructosamine level would lead to a reduction in complication rates. Second, fructosamine was evaluated within 1 month of surgery, and perhaps if it were taken closer to surgery, it may have been a better prognostic marker. Third, there are several factors that may affect serum protein levels, and therefore impact fructosamine levels, which we did not take into consideration. For example, hepatic cirrhosis, protein loosing enteropathies, and other conditions are known to affect plasma protein levels and possibly the level of fructosamine. Finally, fructosamine levels and the threshold used in this study may be affected by differences in labs and reagents used for testing. Future studies are needed to validate the threshold used in this study or suggest a more optimal cut-off level.

This multi-intuitional prospective study supports recent reports on the utility of fructosamine testing prior to total joint arthroplasty. Fructosamine holds major advantages over HbA1c in the preoperative period. We propose that centers without prior experience of using this test, consider adding the test to their screening programs for hyperglycemia, particularly in patients who have fasting glucose greater than 100 mg/dL.
