# Author Correction: Fructosamine is a valuable marker for glycemic control and predicting adverse outcomes following total hip arthroplasty: a prospective multi‑institutional investigation

**DOI:** 10.1038/s41598-021-94867-1

**Published:** 2021-08-02

**Authors:** Noam Shohat, Karan Goswami, Leigham Breckenridge, Michael B. Held, Arthur L. Malkani, Roshan P. Shah, Ran Schwarzkopf, Javad Parvizi

**Affiliations:** 1 Rothman Orthopaedic Institute at Thomas Jefferson University, 125 S 9th St. Ste 1000, Philadelphia, PA 19107 USA; 2 Sackler Faculty of Medicine, Tel Aviv University, Ramat Aviv, Israel; 3 Department of Orthopaedic Surgery, Columbia University, New York, NY USA; 4 Department of Orthopaedics, University of Louisville, Louisville, KY USA; 5 Department of Orthopaedic Surgery, New York University, New York, NY USA

Correction to: *Scientific Reports* 10.1038/s41598-021-81803-6, published online 26 January 2021

The original version of this Article contained an error in Table 2, where in columns “Total cohort (n = 1212)” and “Fasting plasma glucose ≥ 100 mg/dL (n = 423)”, the n values for ‘Normal fructosamine’ and ‘High fructosamine’ were accidently switched. The correct and incorrect heading rows appear below.

Incorrect:

**Table:** 

**Complications**	**Total cohort (n = 1212)**			**Fasting plasma glucose ≥ 100 mg/dL (n = 423)**		
	**Normal fructosamine (n = 54)**	**High fructosamine (n = 1158)**	**p value**	**Normal fructosamine (n = 32)**	**High fructosamine (n = 391)**	**p value**

Correct:

**Table:** 

**Complications**	**Total cohort (n = 1212)**			**Fasting plasma glucose ≥ 100 mg/dL (n = 423)**		
	**Normal fructosamine (n = 1158)**	**High fructosamine (n = 54)**	**p value**	**Normal fructosamine (n = 391)**	**High fructosamine (n = 32)**	**p value**

The original Article has been corrected.

